# Supplementary material for: JNK signaling triggers spermatogonial dedifferentiation during chronic stress to maintain the germline stem cell pool in the Drosophila testis
Source: eLife. 2018 Jul 9;7:e36095. doi: 10.7554/eLife.36095 (PMC6070334; doi:10.7554/eLife.36095)
Supplement: Supplementary file 1. — Data are presented as the mean ± S.E. [file elife-36095-supp1.docx]

| Genotype | Treatment | n | GSCs no. | Relative GSCs no. | Dedifferentiated GSCs no. | % of dedifferentiated GSCS |
| --- | --- | --- | --- | --- | --- | --- |
| *bam-Gal4 > UAS-LacZ* | 0 days | 46 | 10.44 ± 0.32 | 1 ± 0.03 | 0.75 ± 0.03 | 6.99 ± 1.04 % |
|  | 45 days | 64 | 9.42 ± 0.34 | 0.90 ± 0.03 | 2.11 ± 0.44 | 21.56 ± 4.42 % |
|  | 15d. starved | 34 | 6.97 ± 0.41 | 0.67 ± 0.04 | 0.30 ± 0.10 | 4.21 ± 1.47 % |
|  | 15d. starved + 1d. refed | 41 | 7.33 ± 0.38 | 0.70 ± 0.04 | 0.65 ± 0.19 | 7.38 ± 2.16 % |
|  | 15d. starved + 2d. refed | 68 | 8.74 ± 0.26 | 0.84 ± 0.03 | 1.06 ± 0.23 | 12.12 ± 2.65 % |
|  | 15d. starved + 3d. refed | 50 | 9.50 ± 0.35 | 0.91 ± 0.03 | 1.52 ± 0.39 | 14.64 ± 3.67 % |
|  | 15d. starved + 4d. refed | 37 | 10.00 ± 0.43 | 0.96 ± 0.04 | 1.89 ± 0.43 | 17.01 ± 3.85 % |
|  | 15d. starved + 5d. refed | 78 | 9.96 ± 0.31 | 0.95 ± 0.03 | 2.23 ± 0.44 | 19.72 ± 3.66 % |
|  | 15d. starved + 7d. refed | 23 | 10.40 ± 0.40 | 1.00 ± 0.04 | 2.31 ± 0.79 | 21.10 ± 5.68 % |
|  | 15d. starved + 9d. refed | 71 | 9.90 ± 0.30 | 0.95 ± 0.03 | 2.16 ± 0.40 | 19.71 ± 3.56 % |
|  | 45d. mated | 32 | 10.97 ± 6.65 | 1.05 ± 0.05 | 3.47 ± 0.81 | 30.82 ± 6.65 % |
|  | x4 Cycles | 47 | 10.68 ± 0.45 | 1.02 ± 0.04 | 5.03 ± 0.67 | 43.90 ± 7.30 % |
| *bam-Gal4 > UAS-bam* | 0 days | 42 | 7.88 ± 0.32 | 1 ± 0.04 | 0.28 ± 0.26 | 3.30 ± 2.07 % |
|  | 45 days | 45 | 7.06 ± 0.20 | 0.89 ± 0.03 | 0.29 ± 0.21 | 3.60 ± 2.39 % |
|  | 15d. starved | 20 | 5.90 ± 0.52 | 0.74 ± 0.07 | 0 ± 0 | 0.70 ± 0.50 % |
|  | 15d. starved + 3d. refed | 18 | 7.10 ± 0.45 | 0.90 ± 0.05 | 0.09 ± 0.07 | 1.82 ± 1.82 % |
|  | 15d. starved + 5d. refed | 59 | 7.36 ± 0.20 | 0.93 ± 0.03 | 0.10 ± 0.08 | 1.01 ± 0.80 % |
|  | 45d. mated | 22 | 6.42 ± 0.39 | 0.82 ± 0.05 | 0.05 ± 0.05 | 0.88 ± 0.88 % |
|  | x4 Cycles | 39 | 5.44 ±0.34 | 0.69 ±0.04 | 0.13 ± 0.07 | 2.06 ± 1.16 % |
| *bam-Gal4 > UAS-puc* | 0 days | 38 | 7.67 ± 0.20 | 1 ± 0.03 | 0.11 ± 0.05 | 1.39 ± 0.67 % |
|  | 15d. starved + 5d. refed | 47 | 7.26 ± 0.32 | 0.98 ± 0.04 | 0.21 ±0.10 | 3.09 ± 1.50 % |
| *bam-Gal4 > UAS-bsk^K53R^* | 0 days | 44 | 11.16 ± 0.41 | 1 ± 0.04 | 0.82 ± 0.24 | 7.41 ± 2.19 % |
|  | 15d. starved + 5d. refed | 61 | 10.59 ± 0.32 | 0.95 ± 0.03 | 0.93 ± 0.26 | 9.09 ± 2.47 % |
|  |  |  |  |  |  |  |
