# Supplementary material for: JNK signaling triggers spermatogonial dedifferentiation during chronic stress to maintain the germline stem cell pool in the Drosophila testis
Source: eLife. 2018 Jul 9;7:e36095. doi: 10.7554/eLife.36095 (PMC6070334; doi:10.7554/eLife.36095)
Supplement: Supplementary file 2. [file elife-36095-supp2.docx]

| Condition | Marker | n (testes) | n (cells) | GSC type | Negative cells | Positive cells | % of positive cells |
| --- | --- | --- | --- | --- | --- | --- | --- |
| *bam > LacZ –*  4x Cycles | EdU | 82 | 646 | *bam* lineage- | 417 | 108 | 22.64% |
|  |  |  |  | *bam* lineage+ | 169 | 51 | 30.17% |
|  | pH3 | 100 | 1221 | *bam* lineage- | 932 | 75 | 8.05% |
|  |  |  |  | *bam* lineage+ | 189 | 25 | 13.23% |
| *bam > LacZ –*  15 days unmated | pH3 | 64 | 901 | *bam* lineage- | 652 | 42 | 6.44% |
|  |  |  |  | *bam* lineage+ | 195 | 12 | 6.15% |
| *bam > LacZ –*  15 days mated | pH3 | 70 | 788 | *bam* lineage- | 544 | 52 | 9.56% |
|  |  |  |  | *bam* lineage+ | 169 | 23 | 13.61% |
