# Supplementary material for: JNK signaling triggers spermatogonial dedifferentiation during chronic stress to maintain the germline stem cell pool in the Drosophila testis
Source: eLife. 2018 Jul 9;7:e36095. doi: 10.7554/eLife.36095 (PMC6070334; doi:10.7554/eLife.36095)
Supplement: Supplementary file 3. — Data are presented as mean ± S.E.n.a is not applicable [file elife-36095-supp3.docx]

| Genotype | Spermatogenesis stage | Number | Relative to the number of GSCs |
| --- | --- | --- | --- |
| *bam > LacZ –*  0 days  (n = 30) | GSCs | 10.49 ± 0.32 | n.a |
|  | 1-cell cysts | 9.23 ± 0.41 | n.a |
|  | 2-cell cysts | 8.43 ± 0.42 | n.a |
|  | 4-cell cysts | 4.5 ± 0.27 | n.a |
|  | 8-cell cysts | 4.17 ± 0.19 | n.a |
| *bam > bam –*  0 days  (n = 30) | GSCs | 7.27 ± 0.21 | n.a |
|  | 1-cell cysts | 9.10 ± 0.37 | n.a |
|  | 2-cell cysts | 8.00 ± 0.46 | n.a |
|  | 4-cell cysts | 4.63 ± 0.32 | n.a |
|  | 8-cell cysts | 3.90 ± 0.23 | n.a |
| *bam > LacZ –*  4x cycles  (n = 36) | GSCs | 10.83 ± 0.58 | n.a |
|  | 1-cell cysts | 10.08 ± 0.60 | n.a |
|  | 2-cell cysts | 10.69 ± 0.58 | n.a |
|  | 4-cell cysts | 4.94 ± 0.28 | n.a |
|  | 8-cell cysts | 4.41 ± 0.21 | n.a |
| *bam > bam –*  4x cycles  (n = 30) | GSCs | 6.40 ± 0.49 | n.a |
|  | 1-cell cysts | 6.53 ± 0.40 | n.a |
|  | 2-cell cysts | 6.93 ± 0.41 | n.a |
|  | 4-cell cysts | 3.27 ± 0.30 | n.a |
|  | 8-cell cysts | 2.60 ± 0.20 | n.a |
| *bam > LacZ –*  30 days, GFP+ spermatoginia  (dediff.)  (n = 28) | GSCs | 3.89 ± 0.64 | 1 |
|  | 1-cell cysts | 4.07 ± 0.62 | 1.23 ± 0.13 |
|  | 2-cell cysts | 4.00 ± 0.58 | 1.25 ± 0.16 |
|  | 4-cell cysts | 2.14 ± 0.36 | 0.64 ± 0.13 |
| *bam > LacZ –*  30 days, GFP- spermatogonia  (wild type)  (n = 28) | GSCs | 7.39 ± 0.86 | 1 |
|  | 1-cell cysts | 5.71 ± 0.67 | 0.82 ± 0.05 |
|  | 2-cell cysts | 6.18 ± 0.79 | 0.79 ± 0.07 |
|  | 4-cell cysts | 2.71 ± 0.39 | 0.35 ± 0.05 |
|  |  |  |  |
